# Unimproved water and sanitation contributes to childhood diarrhoea during the war in Tigray, Ethiopia: a community based assessment

**DOI:** 10.1038/s41598-023-35026-6

**Published:** 2023-05-13

**Authors:** Akeza Awealom Asgedom, Birhanu Tewoldemedhin Abirha, Askual Girmay Tesfay, Kelali Kaleaye Gebreyowhannes, Hayelom Birhanu Abraha, Gessessew Bugssa Hailu, Mesele Bahre Abrha, Mache Tsadik, Tesfay Gebregziabher Gebrehiwet, Aregawi Gebreyesus, Tilahun Desalew, Yibrah Alemayehu, Afework Mulugeta

**Affiliations:** 1 Department of Environmental Health and Behavioural Sciences, School of Public Health, College of Health Sciences, Mekelle University, Mekelle, Ethiopia; 2 Department of Hygiene and Environmental Health, Tigray Health Bureau, Mekelle, Ethiopia; 3 Department of Parasitology, Tigray Health Research Institute, Mekelle, Ethiopia; 4 Department of Medical Parasitology and Entomology, School of Medicine, College of Health Sciences, Mekelle University, Mekelle, Ethiopia; 5 Department of Reproductive Health, School of Public Health, College of Health Sciences, Mekelle University, Mekelle, Ethiopia; 6 Department of Health Systems, School of Public Health, College of Health Sciences, Mekelle University, Mekelle, Ethiopia; 7 Department of Epidemiology, School of Public Health, College of Health Sciences, Mekelle University, Mekelle, Ethiopia; 8 OXFAM Emergency Response - Tigray Field Office, Mekelle, Ethiopia; 9 Tigray Health Bureau, Mekelle, Ethiopia; 10 Department of Nutrition and Dietetics, School of Public Health, College of Health Sciences, Mekelle University, Mekelle, Ethiopia

**Keywords:** Diseases, Risk factors

## Abstract

Access to water, sanitation, and hygiene (WASH) is a global public health problem. The situation is worst in conflict areas, where people are displaced from their usual homes. Household supply of WASH and the incidence of diarrhoeal disease among children during the war in Tigray are not known or documented. The objective of this study was to investigate the sources of drinking water, sanitation and hygiene practices, and the incidence of diarrhoeal diseases among children during the war in Tigray, Ethiopia. A cross—sectional study was conducted to collect data on selected WASH indicators in six zones of Tigray from August 4-20, 2021. Data were collected from a total of 4381 sample households selected by lottery. Descriptive analysis was performed and the analysed data are presented in tables, figures and explanatory notes. Binary logistic regression was performed to examine the relationship between independent and dependent variables. A total of 4381 households from 52 woredas participated in the study. Approximately 67.7% of the study participants reported that they relied on an improved source of drinking water during the war. Coverage of sanitation, hand washing, and menstrual hygiene during the war was reported as 43.9%, 14.5%, and 22.1%, respectively. The prevalence of diarrhoeal diseases among children was 25.5% during the war. Water source, latrine type, solid waste disposal and health extension worker visits were the significant predictors of the likelihood of diarrhoea in children (*p* < 0.05). The results of the study show that a decrease in services from WASH is associated with a higher prevalence of diarrhoeal disease among children during the war in Tigray. To prevent the high prevalence of diarrhoeal disease among children in war-torn Tigray, Ethiopia, improved access to water and sanitation is recommended. In addition, collaborative efforts are needed to engage health extension workers to provide appropriate promotion and prevention services to war-affected communities in Tigray, Ethiopia. Further comprehensive surveys of households with children over one year of age are recommended to assess access to WASH and the burden of WASH associated diseases.

## Introduction

The issue of water, sanitation, and hygiene (WASH) remains a global public health problem, as outlined in the Sustainable Development Goals (SDGs)^1,2^. However, WASH problems may be more pronounced in populations affected by war and conflict. Globally, millions of people have been displaced or forced to flee their countries due to armed conflict^3^. In such crises, people are at risk of infrastructure collapse, food insecurity, unsafe water, and inadequate water supply and sanitation, particularly affecting women and children^4^. Achieving universal access to safely managed water services by 2030 will require a 23-fold increase in the current rate of progress, a ninefold increase in sanitation, and a fivefold increase in hygiene^2^.

According to the World Health Organization, inadequate WASH contributes to 842,000 deaths in low and middle-income countries, accounting for 58% of all deaths from diarrheal diseases. Unsafe and inadequate drinking-water leads to 502, 000 deaths, inadequate sanitation leads to 280,000 deaths, and inadequate hand washing leads to 297,000 deaths^5^. Each year, approximately 525,000 children under the age of five die from diarrheal diseases^6^. Studies show that the number of under-five deaths indirectly attributable to conflict is three to five times higher than the number of directly attributable deaths^4^.

In Ethiopia, the improvement of WASH services is hampered by various humanitarian situations, such as large-scale internal displacement of people, political tensions, conflicts and mobilization that worsen the security situation in the country^7^. In the pre-war period, various WASH projects were implemented in the Tigray region, such as the One WASH National Program to reduce WASH-related mortality and morbidity^8^.

In November 2020, a drastic war broke out in the Tigray region of Ethiopia^9–11^, resulting in the worst human suffering ever^12,13^. For rehabilitation purposes and evidence-based decision-making, adequate information should be collected on the impact of the war on the condition of WASH facilities and the burden of diarrheal disease in children. However, there is a large gap in knowledge about what has happened to WASH facilities in Tigray and the impact on health. In addition, household sources of drinking water, sanitation and hygiene practices, and the burden of diarrheal disease in children under one year of age as a result of the war have not been documented. Therefore, the objective of this community-based study was to assess the drinking water sources, sanitation facilities, hygiene practices, and prevalence of diarrheal diseases among children during the war in Tigray, i.e., from November 2020 to June 2021.

## Methods

### Study design and setting

A community-based cross-sectional study was conducted in six zones of Tigray, excluding the western zone (for security reasons), to assess drinking water sources, sanitation facilities, hygiene practices, and the prevalence of diarrhoeal diseases among children during the war (from November 2020 to June 2021). Data were collected from August 4 to 20, 2021. Tigray has seven zones and 93 woredas and the study was conducted in 52 woredas randomly selected from six zones. However, 9 woredas of the western zone were not included in the study.

### Study population, sample size and sampling technique

This study was part of a large-scale ‘Key Performance Indicators for Health survey (primarily examining for maternal health, child health, nutritional status, WASH, and civilian injuries) conducted in 52 randomly selected woredas (woreda is an equivalent term for district) of Tigray Regional State in northern Ethiopia. The study is based on the Ethiopian Demographic and Health Survey (EDHS), which serves as the sampling area. Accordingly, four enumeration areas (locally referred to as ‘kebeles’ or ‘tabias’) per selected woreda were considered for the study. In total, there were 208 enumeration areas from 52 woredas. Twenty households in each enumeration area were considered for data collection, resulting in a total sample of 4160 households. However, additional data were collected in some enumeration areas, resulting in a total sample of 4381 households that was used for the analysis. The study followed a multistage sampling procedure (zone, woreda, kebele/tabia and households) and households were selected by lottery. The household heads of the households were the subjects of this study.

### Inclusion and exclusion criteria

The inclusion criteria for the study participants were households with children under one year of age in their household, and the others were excluded. The main purpose of including households with children under one year of age was to collect maternal and child health indicators as part of the large survey of key health care performance indicators.

### Questionnaire development and data collection

A pretested and adapted standardized questionnaire (*Core questions on drinking water, sanitation and hygiene for household surveys: 2018 update*) used for the household WASH survey^14^ was used to collect data from the study households and ask questions on selected key indicators of water supply, sanitation and hygiene to achieve the objective of this study. Data were collected by health extension workers. There was one local supervisor and two data collectors per enumeration area. In addition, one supervisor was assigned to each woreda. Supervisors were selected from College of Health Sciences of Mekelle University, Tigray Health Bureau, and Tigray Health Research Institute to follow and monitor all data collection activities.

### Data quality control

The questionnaire used for data collection was translated from English to Tigrigna and then translated back from Tigrigna to English by another translator to check for language consistency. Prior to the actual data collection, a pre-test of the questionnaire was conducted outside the study site to verify that respondents understood the questions correctly, and if not, to make changes. Data collectors were also trained on the purpose of the study, the method of data collection, ethical principles, and other practical issues. In addition, the questionnaire was reviewed for completeness by direct supervisors on each day of data collection. The data collection instrument used was adapted from a standardized questionnaire to increase the validity of the data.

### Data processing and analysis

Data were entered into Epi Data version 3.1 software and then transferred to SPSS version 25 software for analysis. Descriptive analysis was performed and data were presented using tables, figures, and explanatory notes. Binary logistic regression was performed to examine the association between the independent variables (water, sanitation, hygiene, HEWs visit) and an outcome variable (diarrhoea in children).

### Operational definitions

*Diarrhoea* is defined as loose or watery stools at least three times a day or more frequently than normal for a person.

*During the war* is time from November 4, 2020 to June 2021.

*Pre-war* is the time before November 2020.

*Household* is defined as a group of persons living together in a dwelling with a separate outer door and having a head.

*Improved water sources* are those that by nature of design and construction, are capable of providing safe water. Piped water, boreholes or tube wells, protected dug wells, protected springs, rainwater and packaged or delivered water are improved water sources.

*Unimproved water sources* are those that are unlikely to provide safe water due to their design and construction. Unimproved sources include: unprotected dug wells, unprotected springs, and surface water.

*Improved sanitation facilities* are those designed to hygienically separate human excreta from human contact. These includes wet sanitation technologies such as flush and pour flush toilets connected to sewers, septic tanks or pit latrines, and dry sanitation technologies such as dry pit latrines with slabs and composting toilets.

*Unimproved sanitation facilities* are those that are not designed to hygienically separate human excreta from human contact. These include pit latrines without a slab/open pit, pit latrines without a permanent slab, and easily washable.

*Improved liquid waste management* is liquid waste management with a sink/drain connected to a pit/soak pit.

*Unimproved liquid waste management* is that which is not designed for liquid wastes disposal and is disposed of directly in the open field.

*Improved solid waste management* Solid waste is either through burned or buried.

*Unimproved solid waste management* are those that do not have solid wastes and are simply disposed of in the open field or in the household yard.

*Woreda* is the equivalent name for the district.

*Tabia or kebele* is the lowest administrative unit in a woreda.

### Ethics approval and consent to participate

Ethical approval was obtained from the Mekelle University, College of Health Science Institutional Review Board (Ref. No. MU-IRB 1906/2021). An additional letter of support was obtained from the Tigray Health Bureau to facilitate the study. Written informed consent was obtained from study participants before actual data collection. Confidentiality of the respondents was maintained, and their names were not included in the data. All methods were performed in accordance with the ethical principles of the Declaration of Helsinki.

## Results

### General information

In this community-based study, 4381 households from 52 woredas participated (6 woredas from the southern zone, 6 woredas from the southeastern zone, 3 woredas from the Mekelle zone, 16 woredas from the central zone, 13 woredas from the eastern zone, and 8 woredas from the northwestern zone). All data collected were used for data analysis and interpretation. Of the 4, 381 study participants, 3, 308 (75.5%) of the respondents were female and the remaining 1073 (24.5%) were male. Home visits by health extension workers (HEWs) during the war were reported by only 624 (14.3%) of the study participants.

**Figure 1:**
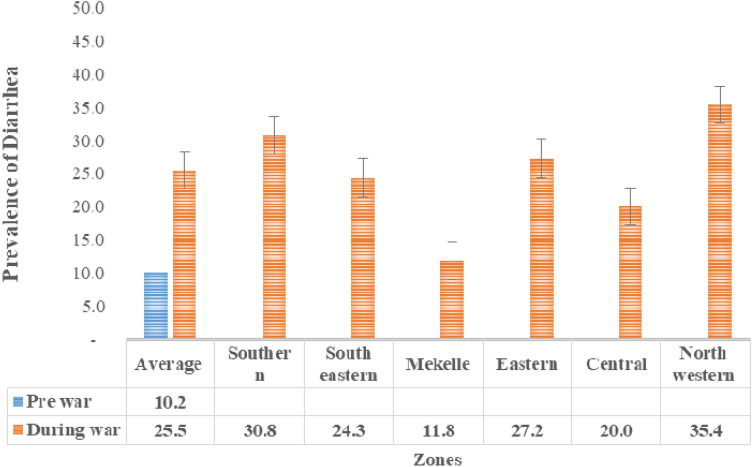
Zonal prevalence of childhood diarrhoea during the war in Tigray.

The overall result on the status of water, sanitation, and hygiene (WASH) shows that the services of WASH were poor during the war. As shown in Table 1, there were zonal differences in the provision of WASH. There was a high prevalence of diarrhoeal disease among children during the war. As shown in Fig. 1, there were also zonal differences in the prevalence of diarrhoeal diseases during the war.

**Table 1:** Pre-war and during war status of water supply, sanitation, hygiene in Tigray (n` = 4381).

Variable	Pre-war regional coverage (%)	During war regional coverage (%)	During war zonal coverage (%)					
			Southern	South Eastern	Mekelle	Eastern	Central	North Western
Improved drinking water sources	84.7^15^	67.7	69.9	68.2	93.6	64.3	69.2	57.4
Functionality of water sources	92.9^16^	27.3	25.1	39.7	11.4	31.9	24.3	25.1
Household based water treatment methods	21.6^17^	17.3	15.1	33.2	28.1	18.5	12.2	10.3
Improved latrine facilities	70.06^15^	43.9	40.8	34.5	87.8	43.1	50.9	22.8
Hand washing facility	47.5^17^	14.5	17.1	9.2	39.8	12	15	7.5
Access for menstrual hygiene materials	ND	22.1	19.4	15.2	39.1	18.4	30.3	12.9
Improved solid waste management practice	52.52^15^	55	50.7	57.5	66.2	54	61.3	44.7
Improved liquid waste management practice	ND	46.2	37.4	43.4	92.1	38.8	61.2	18.1

*ND* No Data.

### Associated factors of diarrhoea in children

The result of the logistic regression model shows that water source, latrine type, solid waste management, and HEWs visit made a statistically significant contribution to the model as shown in Table 2 (*p* < 0.05), while handwashing facility made no contribution to the model (*p* = 0.526). HEWs visit and latrine type were the strongest predictors of the model.

**Table 2:** Logistic regression predicting likelihood of diarrhoea in children.

Variable		Diarrhoea		COR	95% C.I. for COR	*p* value	AOR	95% C.I. for AOR	*p* value
		Yes n (%)	No n (%)						
Water source (N = 4319)	Improved	639 (21.9)	2279 (78.1%)	1.69	1.47–1.95	< 0.001	1.42	1.21–1.65	< 0.001
	Unimproved	452 (32.3%)	949 (67.7%)						
Functionality of water source (N = 4316)	Yes	281 (23.8%)	902 (76.2%)			0.087*			
	No	824 (26.3%)	2309 (73.7%)						
Household level water treatment methods (N = 4254)	Yes	173 (23.4%)	567 (76.6%)			0.113*			
	No	920 (26.2%)	2594 (73.8%)						
Type of latrine (N = 4305)	Improved	350 (18.5%)	1537 (81.5%)	1.97	1.71–2.28	< 0.001	1.72	1.45–2.03	< 0.001
	Unimproved	751 (31.1%)	1667 (68.9%)						
Solid waste management (N = 4005)	Improved	147 (20.8%)	559 (79.2%)	1.42	1.16–1.73	< 0.001	1.30	1.05–1.61	0.016
	Unimproved	897 (27.2%)	2402 (72.8%)						
Liquid waste management (N = 1859)	Improved	206 (17%)	1005 (73%)			0.087*			
	Unimproved	131 (20.2%)	517 (79.8%)						
Hand washing facility (N = 4035)	Yes	105 (17.8%)	484 (82.2%)	1.71	1.37–2.14	< 0.001	1.08	0.84–1.40	0.526
	No	935 (27.1%)	2511 (72.9%)						
HEWs visit (N = 4337)	Yes	96 (15.8%)	513 (84.2%)	1.98	1.58–2.50	< 0.001	1.91	1.44–2.51	< 0.001
	No	1011 (27.1%)	2717 (72.9%)						
Constant					0.09		< 0.001		

*N* Sample size; *HEWs* Health Extension Workers; *n* Frequency; *COR* Crude Odds Ratio; *AOR* Adjusted Odds Ratio; *Only Chi-square test performed.

## Discussions

The main finding of the present study shows a high prevalence of diarrhoeal disease among children during the war in Tigray, Ethiopia. The majority of households relied on unimproved drinking water sources and unimproved sanitation facilities. Hygiene practices were also poor, with few menstrual hygiene materials available during the war in Tigray, Ethiopia.

The prevalence of diarrhoeal disease in children under one year of age was higher in the present study (25.5%) than in the pre-war survey (10.2%)^17^. Our results are consistent with those of other studies conducted in flood prone settlements and conflict^18,19^. However, the result of the present study is higher than that of other studies conducted in community^20–22^. The difference could be explained by the war in our study, in which all services were affected compared with other studies conducted under normal circumstances.

A higher prevalence of diarrhoeal disease in children was found in households not visited by HEWs than in households visited by HEWs. The frequency of home visits by HEWs was lower during the war than in the pre-war study^15^. The Health Extension Program is a flagship program of the Government of Ethiopia, launched in 2003 Gregorian calendar under the Health Sector Development Program (HSDP –II), targeting households to provide health promotion and preventive services in communities at the grassroots^23^. To this end, more than 38,000 HEWs have been trained and deployed throughout the country (two HEWs in each kebele/tabia)^24^ to provide health promotion and preventive services to the population. Most of these services are focused on hygiene and environmental sanitation programs. Although HEWs play an important role in reducing the burden of diarrhoeal diseases^25^, most HEWs have not been able to carry out their work because they have either been resettled, migrated, or died due to the war in Tigray^11^. Evidence suggests that scaling up health extension programs reduces child morbidity and mortality due to diarrhoeal diseases^24^, but the low frequency of household visits by HEWs makes it difficult to follow up on hygiene and environmental sanitation programs and other health packages in the community. As a result, the reported high prevalence of diarrhoeal disease among children may be exacerbated by the neglected role of HEWs in community health promotion and disease prevention.

A large number of households reported relaying on unimproved water sources, which resulted in a higher prevalence of diarrhoeal disease among children in the present study. This finding is consistent with the results of another study^26^, in which unimproved water sources were one of the identified predictors of diarrhoeal disease. The percentage of households relying on unimproved water sources was higher than the 2018 regional socioeconomic survey^15^. This study also showed that only a quarter of the water sources were reported to be functional at the time of the study and the rest were non-functional. This could be due to direct damage to the water source, lack of maintenance, and lack of budgetary resources. The non-functioning of water sources could force people to search for unimproved water sources, leading to morbidity and mortality. War-related damage to various water sources is believed to affect the functionality of water sources in Tigray. Damage to water sources could indicate the use of water blockades as a weapon of war, as observed elsewhere^27^. It was found that only 17.3% of respondents use water treatment methods in the household, indicating that the majority of households still do not have water treatment facilities that could lead to waterborne diseases. Studies have shown that effective and consistent use of household water treatment and safe storage reduces waterborne diseases such as diarrhoeal diseases^28^ by 28% to 45% depending on the type of water supply^5^. However, this study only examined sources of drinking water during the war. It did not examine household access to drinking water, which could be a potential research topic for the future.

The prevalence of diarrhoeal disease was higher in households that relied on unimproved sanitation. The provision of improved sanitation facilities was lower during war than in a 2018 survey^15^. It is well documented that the lack of improved sanitation leads to diarrhoeal disease^26,29,30^. Provision of improved sanitation and safe water has been shown to be a successful method of improving maternal and new-born health in conflict situations^4^.

The percentage of hand washing practice was 14.5%, lower than the pre-war figure of 47.5%^17^. In low- and middle-income countries, promotion of hand washing resulted in a 30% decrease in diarrhoeal disease among children (up to 15 years of age)^31^. However, the higher prevalence of diarrhoeal disease among children may be due to poor WASH services in war-torn Tigray. Improved water, sanitation, and hygiene measures have been shown to reduce the burden of diarrhoeal diseases in children^29,32–37^.

Access to menstrual hygiene materials was reportedly available to only 22.1% during the war. Menstrual hygiene is critical as lack of access to menstrual hygiene materials negatively impacts the health, well-being and dignity of women and girls^2^. The lack of improved water supply, sanitation, and access to menstrual hygiene materials in Tigray burdens women and girls and impacts their health, dignity, and well-being.

### Strength and limitation of the study

This study has its own strengths and limitations. The large sample size, covering most woredas in six zones of Tigray, will increase the validity of the results. The large sample size reduces the sampling error as it increases the representativeness. In addition, the majority of respondents were female, which provides relevant information about WASH and the prevalence of diarrhoeal disease among children. The activities are also the burden of women and children due to the socially constructed task assignment of women. The use of a pretested, standardized questionnaire and the inclusion of supervisors should also increase the validity of the results. To our knowledge, this is the first study conducted to assess WASH after the war in Tigray, Ethiopia.

However, the study also has its own limitations. The results of this study are based on a cross-sectional design which has the limitations of cross-sectional studies. It is inconclusive for a cause and effect relationship between WASH and diarrhoeal disease in children. Respondent sociodemographic data were not collected for this study for fear of study participant willingness. These sociodemographic and other factors were not considered in this study, which could be confounding factors for diarrhoeal illness in children. The exclusion of Western Tigray from this study could also affect the nature of the overall results. This study also did not examine access to drinking water supplies. The prevalence of diarrhoeal disease in children older than one year age was not examined in this study. In addition, the prevalence of diarrhoeal disease in children after the eight- month war (particularly during the siege in Tigray) was not studied and is not known. This is believed to affect the services WASH and the burden of diarrhoeal diseases and other WASH related diseases in the community.

## Conclusions

The overall result of the study shows that during the war in Tigray, Ethiopia, the frequency of home visits by health extension workers was low, water and sanitation services were not improved, and the prevalence of diarrhoeal diseases among children was high. In addition, there were zonal differences in the selected WASH indicators and the prevalence of diarrhoeal diseases in children.

Based on the findings of this study, better access to improved water and sanitation services is recommended to prevent the high prevalence of diarrhoeal diseases among children in war-torn Tigray, Ethiopia. Collaborative efforts are also needed to engage health extension workers to provide promotive and preventive services to war affected communities in Tigray, Ethiopia. Further comprehensive surveys of households with children over one year of age are recommended to assess access to WASH and the burden of WASH associated diseases.

## Data Availability

The datasets used and/or analysed during the current study are available from the corresponding author on reasonable request.

## Abbreviations

AOR: Adjusted odds ratio
CI: Confidence interval
EDHS: Ethiopian demographic health survey
HEWs: Health extension workers
HH: Household
HSDP: Health sector development programme
MU-IRB: Mekelle University Institutional Review Board
ND: No data
Ref.No.: Reference number
SDGs: Sustainable development goals
SPSS: Statistical package for social science
UNICEF: United nation children’s fund
WASH: Water, sanitation and hygiene
WHO: World health organization
